# Round the Hospitals

**Published:** 1919-07-26

**Authors:** 


### ROUND THE HOSPITALS.

Under the recommendations of the committee appointed to consider the question of war medals, nurses become eligible for both the Victory Medal and the British War Medal. The Victory Medal is the decoration of all the Allies, and it is estimated that four million Britons are entitled to it. It is to be of bronze, with a ribbon identical for all countries; on one side a winged figure of Victory, on the reverse "The Great War for Civilisation," in the language of the respective countries. It is to be assigned to all those who "entered a theatre of war," in the strength of any military unit, irrespective of their duties. It has been judged impossible to issue a war workers' medal such as

(Continued on page 434.

**ROUND THE HOSPITALS**—*(continued from p. 431)*.
was forecasted by Lord Kitchener early in 1915, because of the impracticability of distinguishing "war work" from other services indispensable to the national welfare. The British War Medal, which will be of silver, will be awarded to all who have entered a theatre of war on duty attached to an organised force. Women belonging to any independent organisation recognised by the Naval, Military, or Air Forces, will receive the British Medal only.

As time goes on it becomes abundantly clear that so far, from the British Women's Hospitals Committee having said too much about the needs of nurses, the facts about invalided Army sisters are not yet grasped by the public. One of the sisters lately attached to Q.A.I.M.N.S., writes to the *Morning Post* that many of her comrades "having done three, four, or five years' service abroad, now find themselves invalided and permanently unfit." A temporary pension at the rate of £30 a year is granted in such cases for six months, with leave to apply for a further extension at the end of that time. The disabled nurse may also obtain £2 2s. a week for a period of six weeks from the Pensions Board, if her doctor can certify that she needs treatment in order to take up some other form of work. But, of course, where, as so often happens, the sister has no home of her own, six weeks is not nearly enough. Each case needs individual consideration, and there should be a special department of the Pensions Board where the requirements and disabilities of Army nurses can be promptly and sympathetically dealt with.

Mr. C. E. Leonard Lyle, M.P. for Stratford, who, it will be remembered, took an active part in the debate on the Nurse Registration Bill, has expressed his intention of celebrating peace by surrendering his first year's salary as Member to provide comforts for nurses. Mr. Lyle is Chairman of Queen Mary's Hospital, Stratford. He is one of the members chosen by the College of Nursing to uphold nurses' interests in the House of Commons, and he strongly supported the proposal that the Government, after impartial inquiry, should bring in a Registration Bill of their own.

One of the first acts of the newly formed Ministry of Health has been to draw up, in consultation with the Board of Education, a syllabus of training for health visitors. Provision is made for two types of training. The full course of two years is intended for ordinary students of a minimum age of eighteen. For trained nurses the course is shortened to one year. It is certain that girls of twenty, even if they take additional training in midwifery and other branches of work, will not be fit for the full responsibilities of health visitors, but they will be very useful in subordinate posts connected with welfare work. The courses are to comprise instruction in elementary physiology, as the only reliable basis for instruction in the other subjects, in cooking, household economy, and domestic arrangements. A course of hygiene is contemplated, infant and child welfare are to be fully dealt with, and elementary economics and social problems are to be studied.

The demand for institutional training for the embryo health visitor is imperative. Where practicable, these students are to spend part of their training period in a maternity or children's hospital or the like. How far the voluntary hospitals will be able to co-operate in this scheme depends on the length of time the students are willing to give to mastering their duties. It is much to be regretted, in our judgment, that so short a period as two years should have been laid down by the Ministry of Health as adequate training for girls of eighteen in the difficult profession of health visiting. Since there is to be a special examining body authorised to issue diplomas to students who have completed an approved course and passed an examination, there is ground for hope that experience will speedily prove the need for prescribing the longer term of training.

An interesting feature at the King's Investiture on July 12 was the bestowal of the Commandership of the British Empire Order (Military Division) on some of the overseas matrons. Miss Mabel Thurstan, Matron-in-Chief of the New Zealand Army Nursing Service, was one of the recipients of the Order, and with her were Miss Ethel Davidson and Miss Ethel Gray, both matrons of the Australian Army Nursing Service. Miss Altamont Smythe, Matron of the British Red Cross Society, was made Officer of the Order (Civil Division). At the Investiture held on July 17, there were no recipients of the Royal Red Cross, First Class. The King conferred the Second Class of the Order on Sister Elizabeth White, Australian A.N.S., and on Mrs. Louise Scott-Bamford, Miss Mabel Capper, and Mrs. Florence Spratt, V.A.D.

On another page we announce the result of the examinations for the College of Nursing Studentships, with the names and training of the successful candidates, and the circumstances which have led to eight instead of five studentships being available. We publish, too, the awards and conditions of the Cowdray Studentships endowed by the Viscountess Cowdray. The Council of the Nightingale Fund has also awarded three scholarships, presented to nurses holding the certificate of the Nightingale Training School, St. Thomas's Hospital, to Miss Theodora West-Watson, Miss Gladys V. Hillyers, Miss Mary E. G. Milne. All the studentships and scholarships provide for one year's course in social and household science at King's College for Women, qualifying the candidates for the higher administrative and educational posts in the nursing profession. We congratulate the eleven successful ladies to whom the College, the Cowdray, and the Nightingale Studentships have been respectively awarded, and shall watch their future careers with interest. The whole nursing profession will be grateful to Lady Cowdray for her strenuous service on behalf of all nurses, and for her latest act of splendid generosity. She is indeed one of the greatest and best friends British nurses have ever had.

